# Factors correlated with neuropathic pain among industrial workers in Vietnam: a multi-site cross-sectional study

**DOI:** 10.3389/fpubh.2023.1007483

**Published:** 2023-08-11

**Authors:** Tham Thi Nguyen, Huong Van Nguyen, Hai Minh Vu, Ryan G. Chiu, Quang Nhat Nguyen, Thao Thi Phuong Nguyen, Long Hoang Nguyen, Anh Kim Dang, Khanh Nam Do, Tu Huu Nguyen, Vu Nguyen, Anh Toan Ngo, Tung Hoang Tran, Carl A. Latkin, Cyrus S. H. Ho, Roger C. M. Ho

**Affiliations:** ^1^Institute for Global Health Innovations, Duy Tan University, Da Nang, Vietnam; ^2^Faculty of Nursing, Duy Tan University, Da Nang, Vietnam; ^3^Department of Neuroscience, Hanoi Medical University, Hanoi, Vietnam; ^4^Department of Trauma and Orthopaedic, Thai Binh Medical University Hospital, Thai Binh, Vietnam; ^5^College of Medicine, University of Illinois at Chicago, Chicago, IL, United States; ^6^Université Claude Bernard Lyon 1, Villeurbanne, France; ^7^Department of Public Health Sciences, Karolinska Institutet, Stockholm, Sweden; ^8^Institute for Preventive Medicine and Public Health, Hanoi Medical University, Hanoi, Vietnam; ^9^Vietnam Young Physician Association, Hanoi, Vietnam; ^10^Department of Surgery, Hanoi Medical University Hospital, Hanoi, Vietnam; ^11^National Hospital of Obstetrics and Gynecology, Hanoi, Vietnam; ^12^Institute of Orthopedic and Trauma Surgery, Vietnam–Germany Hospital, Hanoi, Vietnam; ^13^Bloomberg School of Public Health, Johns Hopkins University, Baltimore, MD, United States; ^14^Department of Psychological Medicine, Yong Loo Lin School of Medicine, National University of Singapore, Singapore, Singapore; ^15^Institute for Health Innovation and Technology (iHealthtech), National University of Singapore, Singapore, Singapore

**Keywords:** neuropathic pain, industrial workers, spinal pain, osteoarthritis pain, work environment, occupational

## Abstract

**Introduction:**

Neuropathic pain is a debilitating condition resulting from various etiologies such as diabetes, multiple sclerosis, and infection, and is associated with decreased quality of life, poor health outcomes, and increased economic burden. However, epidemiological studies on neuropathic pain have been largely limited in Vietnam.

**Methods:**

A cross-sectional study was conducted on adult Vietnamese industrial workers across three manufacturing plants. Demographic, socioeconomic, occupational and health data were collected. Prevalence of neuropathic pain was assessed using the Douleur Neuropathique 4 (DN4) scale. Regression modeling was utilized to identify predictors of pain.

**Results:**

Among 276 workers, 43.1 and 24.3% reported that they had suffered from spinal pain and osteoarthritis pain, respectively. In terms of work conditions, people maintaining constant posture when working from 30 to 60 min (OR = 3.15, 95% CI = 1.07; 9.29), or over 60 min (OR = 2.59; 95% CI = 1.12; 5.98) had a higher risk of suffering from spinal pain. People who worked in conditions lacking adequate lighting and with exposures to toxic chemicals were also likely to be suffering from osteoarthritis pain with OR = 4.26, 95% CI = 1.02; 17.74 and Coef. = 1.93; 95% CI = 1.49; 2.50, respectively. Regular health examinations and higher expenditure for healthcare were correlated with a lower prevalence of neuropathic pain.

**Discussion:**

These results may inform the adoption of pain screening and other programs that increase health care access for this population, as well as more stringent occupational health and safety standards.

## Introduction

Neuropathic pain refers to pain arising from a primary lesion or dysfunction of somatosensory neuronal pathways themselves, which is contrary to the more common and well-recognized nociceptive form of pain where systems remain intact and relay noxious peripheral stimuli secondary to a wide array of underlying pathologies (e.g., arthritis, trauma, infection, toxins) (1). Neuropathic pain is often associated with significant decrease in quality of life, work productivity, health status, and longevity (2–4). Specifically, the long-term, often life-long, impacts of neuropathic pain include sleep disorders, heightened depression symptoms, which are often defined as the “TRIAD of pain” (5). The most common forms of neuropathic pain include spinal pain and osteoarthritis pain, whose lifetime prevalence worldwide were reported as 53 to 80% and 31 to 45%, respectively (6, 7). Globally, neuropathic pain conditions affect roughly 13% of the total workforce and result in a mean of 4.6 h loss of productivity per week, according to the American Productivity Audit (8). While no official record **exists** in Vietnam, the total economic burden of neuropathic pain has been estimated to be over $27,000 per patient annually in the United States and approximately (9).

The prevalence of neuropathic pain worldwide ranges from 3 to 17% (10). However, this figure is not entirely representative, especially of low- and middle-income countries as data available are completely limited to populations from Europe and the United States (11). Various environmental and lifestyle factors have been found in correlation with the development of neuropathy pain, including but not limited to stress, anxiety, trauma, and depression, all of which are more prevalent in populations of lower socioeconomic status (9, 12, 13). Likewise, protective factors, such as social support, active coping, acceptance, and self-efficacy are often less accessible within these populations (14). As prevalence of neuropathic pain and its associated factors differ across nations, regions, and occupations, rigorous evidence is necessary to acquire a comprehensive understanding of this global health issue.

In general, a lack of occupational health and safety standards, as well as lack of health screening for factory workers are common in rising industrial regions, which may create environmental conditions that exacerbate the prevalence and incidence of neuropathic pain (15). In recent years, Vietnam has emerged as a rapidly industrializing region, indicated by extensive expansion of the manufacturing sector (16). As a result, risk factors for neuropathic pain have also been amplified within the Vietnamese population and especially among industrial workers. Nonetheless, epidemiological studies on neuropathic pain have been largely limited, if not nonexistent, in Vietnam. Therefore, this study is among the first to evaluate the prevalence and correlated factors of neuropathic pain among workers in large factories in Vietnam. The results of this study provide valuable insights for health policy practices to develop protection measures for this vulnerable population.

## Materials and methods

###  Study design, and sampling method

A cross-sectional study of workers in three factories in Hanoi and Bac Ninh provinces was conducted between July and September 2018. Hanoi and Bac Ninh are the two technology development hotspots within the largest industrial zones in Northern Vietnam. In this study, we purposively selected three factories, including electronic components manufacturers (A Company), producing vehicle accessories (B Company) and manufacturing automotive electrics, home electronics, telecommunications devices, and control devices (C Company). At the time of collecting data, the total number of workers in each company was ~2,000, 1,150, and 6,000. The eligible criteria for selecting participants in the study included (1) at least 18 years; (2) being workers and having current labor contracts with the three aforementioned factories for at least 6 months; (3) agreeing to participate in the study and sign the written informed consent; and (4) having the capacity to communicate with the data collectors. In this study, we excluded participants who suffered from severe illnesses such as malignancy, compression fractures, diffuse pains (such as fibromyalgia or ankylosing spondylitis), chronic alcoholism or substance abuse, serious psychiatric disorders, and any other clinically relevant disease preventing neuropathy assessment or accurate understanding of the questionnaires (cognitive impairment, dementia, etc.). Furthermore, people with higher positions than workers (such as managers, directors, etc.) were also excluded from the study.

In this study, a convenience sampling method was used to recruit participants. A population proportion estimation formula was applied to calculate the sample size in this study, with the confidence level was 95%, the expected spinal pain proportion was 27.5% [according to previous study in the US (17)], and relative precision was 0.20. After calculating, the necessary sample size was 278 workers. To prevent incomplete responses or dropout, 5% was added to the sample size, resulting in a total of 292 workers who were invited to participate in the study. At the end of the data collection duration, 276 workers agreed to participate in this study, with a response rate of 94.5%.

### Measurement and instrument

A structured questionnaire consisting of five main components was developed to collect data, including (1) socio-demographic characteristics, (2) health status, (3) health behaviors, (4) working conditions, and (5) neuropathic pain characteristics. Before the interview process, this questionnaire was piloted in 20 people to test the language, logical order of each question, and modify the question with unclear meanings. During the data collection period, once participants agreed to participate in this study, they were fully informed about the purpose of the study, benefits, and drawbacks. Participants also signed written informed consents acknowledging their rights and protection of their confidentiality. After that, a face-to-face questionnaire was conducted for 15 to 20 min by investigators who were well-trained to use questionnaires. Additionally, the interview took place in a closed room, to ensure privacy and limit outside influences.

### Variables

#### Outcome variables

##### Neuropathic pain

The Douleur Neuropathique 4 (DN4) questionnaire was developed to screen for symptoms and signs of neuropathic pain (18). The original version of this scale was developed and validated in French (19). In this study, a DN4 questionnaire with nine items according to the publication by Giorgio Cruccu et al. and Michael et al. was applied (20, 21) to assess pain description characteristics (burning, painful cold, electric shocks), and associated abnormal sensations (tingling, pins, and needles, numbness, itching) (20–22). For each item of DN4, 1 point is respectively with each positive item and 0 point is respectively with each negative item. A cutoff score of 4 was utilized for the detection of neuropathic pain. Until now, the DN4 scale had been validated by several previous studies and showed a high level of sensitivity and specificity of 82.9 and 89.9%, respectively (19, 22).

##### Spinal pain and osteoarthritis pain

To explore the information about spinal pain and Osteoarthritis pain status among participants, two questions were developed, including 1) “Have you ever felt spinal pain during the last 3 months?”, and “Have you ever felt osteoarthritis pain during the last 3 months?”, with two answer options (Yes/No).

#### Covariates

##### Socio-demographic characteristics

Respondents self-reported information about age (years), gender (male/female), level of education (under high school/high school/tertiary or higher), marital status (living with spouse, partner/other), number of children (none/one/two/three or above), and amount of expenditure for health care per year (USD).

##### Health status

To assess health status among participants, they were asked to report information about acute symptoms and chronic diseases by using following questions: (1) “Have you ever experienced any acute symptoms during the last 4 weeks?” (Yes/No), and (2) “Have you ever been diagnosed with any chronic diseases during the last 3 months?” (Yes/No). These acute symptoms included headache, allergy, constipation, cough/sore throat, etc. Meanwhile, the chronic diseases consisted conditions such as of hypertension, cardiovascular, diabetes, cancer, asthma, epilepsy/psychiatry, stomach/digestive, chronic obstructive pulmonary disease, etc.

##### Health behaviors

Participants were then classified into three types including current, former, and never-smokers. Current smokers were classified if for the whole lifetime the participants smoked at least 100 cigarettes and smoked in the 30 days previously. Participants who were former smokers were defined if they smoked at least 100 cigarettes for the whole of their lifetime and had not smoked in 30 days ago. Those who had not smoked and had never smoked 100 cigarettes were considered never-smokers. Furthermore, the question “Have you ever consumed alcohol?” was developed to explore the alcohol consumption status among participants. For this question, if participants reported “Yes,” they would be classified into the “alcohol consumption group.”

##### Working conditions

Participants self-reported the length of their occupational history (in years), the number of working hours per day, whether they utilize protective equipment (e.g., helmets, goggles, etc.), periodic health examinations in the company (yes/no), hazardous occupational exposures [loud noise, dust, hot temperatures, poisonous gas, low-light settings, wet environments, toxic chemicals, accident-prone settings (yes/no)], and the longest duration of time spent maintaining a constant posture in a typical workday (<30 min/30–60 min/over 60 min).

###  Statistical analysis

Both descriptive and analytical statistics were used to address the main aims of the study by STATA version 16. In this study, Skewness and kurtosis test (sktest) was utilized to evaluate the distributions of quantitative variables. The descriptive analysis was applied to all variables. For quantitative variables, we presented the mean and standard deviation (SD) values for normal distribution variables and the median and interquartile range (IQR) values for non-normal distribution variables. While for categorical variables, we were presented as frequencies and percentages. Comparison of the differences between spinal pain group, osteoarthritis pain group and other variables, Chi-square test, and Rank-sum test was applied. In this study, the multilevel mixed-effects logistic regression models were applied to identify factors associated with spinal pain, and osteoarthritis pain, while the multilevel mixed-effects linear regression model was used to indicate factors related to neuropathic pain (DN4 score) among participants. The potential variables for full models included socio-economic status, health status, health behavior, and work conditions. A *p*-value (p) <0.05 was considered statistically significant.

## Results

Table 1 describes the demographics and health status characteristics among industrial workers in this study. The results showed that the percentage of people who suffered from spinal pain was 43.1%, osteoarthritis pain was 24.3%, and both spinal pain and osteoarthritis pain were 16.7%. In the groups of people who suffered spinal pain or osteoarthritis pain or both kinds of pain, the percentage of participants being male, having a tertiary or higher level of education, living with a spouse/partner, and having never smoked were higher than people without suffered pain. Furthermore, compared to people who have not suffered spinal pain or osteoarthritis pain or both kinds of pain, the percentage of respondents having acute symptoms, chronic diseases, and alcohol consumption in groups of people who suffered pain was significantly higher with p < 0.05. Besides, the amount of expenditure for healthcare per year in the group of people suffering from spinal pain was significantly higher than in the group of people who have not suffered ($227.3 ± 309.4 vs. $112.2 ± 113.9) with *p* < 0.05.

**Table 1:** Demographics characteristics and health status among participants.

**Characteristics**	**Spinal pain**					**Osteoarthritis pain**					**Spinal pain and osteoarthritis pain**				
	**No**		**Yes**		***p*** **-value**	**No**		**Yes**		***p*** **-value**	**No**		**Yes**		***p*** **-value**
	***n***	**%**	***n***	**%**		***n***	**%**	***n***	**%**		***n***	**%**	***n***	**%**	
**Total**	157	56.9	119	43.1		209	75.7	67	24.3		230	83.3	46	16.7	
**Name of the company (*****n*** = **276)**															
A	58	36.9	22	18.5	0.002	72	34.4	8	11.9	0.002	78	33.9	2	4.3	<0.001
B	27	17.2	20	16.8		32	15.3	15	22.4		40	17.4	7	15.2	
C	72	45.9	77	64.7		105	50.2	44	65.7		112	48.7	37	80.4	
**Gender (*****n*** = **274)**															
Male	25	16.0	22	18.6	0.569	34	16.4	13	19.4	0.574	37	16.2	10	21.7	0.366
Female	131	84.0	96	81.4		173	83.6	54	80.6		191	83.8	36	78.3	
**Level of education (*****n*** = **274)**															
Under high school	17	11.0	8	6.7	0.324	20	9.7	5	7.5	0.761	23	10.1	2	4.3	0.234
High school	98	63.2	73	61.3		130	62.8	41	61.2		144	63.2	27	58.7	
Tertiary or higher	40	25.8	38	31.9		57	27.5	21	31.3		61	26.8	17	37	
**Marital status (*****n*** = **276)**															
Living with spouse/partner	144	91.7	118	99.2	0.005	197	94.3	65	97	0.371	217	94.3	45	97.8	0.326
Other	13	8.3	1	0.8		12	5.7	2	3		13	5.7	1	2.2	
**Number of children (*****n*** = **263)**															
None	6	4.1	6	5.1	0.887	9	4.5	3	4.7	0.988	9	4.1	3	6.7	0.758
One	22	15.1	20	17.1		31	15.6	11	17.2		35	16.1	7	15.6	
Two	94	64.4	75	64.1		129	64.8	40	62.5		139	63.8	30	66.7	
Three or above	24	16.4	16	13.7		30	15.1	10	15.6		35	16.1	5	11.1	
**Health status and health behaviors**															
**Having acute symptoms (*****n*** **=** **276)**	69	43.9	75	63.0	0.002	101	48.3	43	64.2	0.024	111	48.3	33	71.7	0.004
**Having chronic conditions (*****n*** **=** **276)**	56	35.7	58	48.7	0.029	76	36.4	38	56.7	0.003	86	37.4	28	60.9	0.003
**Having alcohol consumption (*****n*** **=** **266)**	23	15.2	22	19.1	0.01	31	15.4	14	21.5	0.253	33	14.9	12	26.7	0.056
**Current smoking status (*****n*** = **251)**															
Never smokers	127	89.4	91	83.5	0.169	160	86.5	58	87.9	0.92	180	87.4	38	84.4	0.858
Former smokers	5	3.5	10	9.2		11	5.9	4	6.1		12	5.8	3	6.7	
Current smokers	10	7.0	8	7.3		14	7.6	4	6.1		14	6.8	4	8.9	
	**Mean**	**SD**	**Mean**	**SD**	***p*** **-value**	**Mean**	**SD**	**Mean**	**SD**	***p*** **-value**	**Mean**	**SD**	**Mean**	**SD**	***p*** **-value**
**Age (years) (*****n*** **=** **273)**	35.9	4.8	35.7	4.1	0.809	35.7	4.4	36.4	4.9	0.396	35.9	4.7	35.7	3.2	0.82
**Expenditure for health care per year (USD) (*****n*** **=** **272)**	112.2	113.9	227.3	309.4	<0.001	165.6	248.9	162.9	180.6	0.896	160.9	239.2	182.8	198.8	0.574

The working conditions of participants are presented in Table 2. Participants self-reported that noise, dust, and heat were the most common exposure factors in work among workers, while the opposite was true for wet environments. Furthermore, the people who reported spinal pain or osteoarthritis pain, or both types of pain were also more likely to self-report several workplace exposures such as hot, lack of light, toxic chemicals, and working in an accident-prone environment than those who reported no pain. This was true for the spinal pain, osteoarthritis pain, and both spinal pain and osteoarthritis pain with *p* < 0.05. Among participants self-reported that hey suffered spinal pain, osteoarthritis pain, and both spinal pain and osteoarthritis pain, above half of those reported that they were maintaining constant posture over 60 min when working, and these figures are significantly higher than the no pain group. The mean years of working and working hours per day in the spinal pain group were 10.3 ± 4.1 years, and 8.4 ± 1.2 h, higher than no spinal pain group with 9.7 ± 3.6 years, and 8.2 ± 0.6 h, respectively. For the osteoarthritis pain group, the mean years of working (10.1 ± 3.5 vs. 9.9 ± 3.9 years), as well as working hours (8.4 ± 1.1 vs. 8.2 ± 0.8 h) of them were higher than those no pain group. And for the people who suffered both spinal pain and osteoarthritis pain group, the mean years of working (10.1 ± 3.3 vs. 8.5 ± 1.1 years) and working hours of them also higher than people in no spinal pain group.

**Table 2:** Working conditions of participants.

**Characteristics**	**Spinal pain**					**Osteoarthritis pain**					**Spinal pain and osteoarthritis pain**				
	**No**		**Yes**		***p*** **-value**	**No**		**Yes**		***p*** **-value**	**No**		**Yes**		***p*** **-value**
	***n***	**%**	***n***	**%**		***n***	**%**	***n***	**%**		***n***	**%**	***n***	**%**	
**Exposure factors (Yes) (*****n*** = **272)**															
Noise	114	74.5	94	79.0	0.387	155	74.9	53	81.5	0.27	167	73.9	41	89.1	0.026
Dust	70	45.8	67	56.3	0.084	99	47.8	38	58.5	0.135	109	48.2	28	60.9	0.118
Hot	77	50.3	80	67.2	0.005	113	54.6	44	67.7	0.062	122	54.0	35	76.1	0.006
Poisonous gas	52	34.0	51	42.9	0.135	75	36.2	28	43.1	0.321	79	35.0	24	52.2	0.028
Lack of light	9	5.9	16	13.4	0.032	14	6.8	11	16.9	0.013	16	7.1	9	19.6	0.008
Wet	14	9.2	12	10.1	0.795	18	8.7	8	12.3	0.388	19	8.4	7	15.2	0.152
Toxic chemicals	45	29.4	53	44.5	0.01	67	32.4	31	47.7	0.025	72	31.9	26	56.5	0.001
Working in an accident-prone environment	16	10.5	25	21.0	0.016	26	12.6	15	23.1	0.039	29	12.8	12	26.1	0.022
**The amount of time maintaining constant posture when working (*****n*** = **252)**															
Less than 30 min	61	43.3	25	22.5	0.003	75	39.7	11	17.5	0.006	78	37.9	8	17.4	0.030
30–60 min	18	12.8	21	18.9		27	14.3	12	19.0		30	14.6	9	19.6	
Over 60 min	62	44.0	65	58.6		87	46	40	63.5		98	47.6	29	63.0	
**Regular health examination in company (*****n*** **=** **261)**	143	94.7	103	93.6	0.715	187	94.9	59	92.2	0.414	206	94.5	40	93.0	0.705
**Equipped with labor protection (*****n*** **=** **272)**	153	99.4	118	100.0	0.381	206	99.5	65	100.0	0.575	226	99.6	45	100.0	0.656
	**Mean**	**SD**	**Mean**	**SD**	***p*** **-value**	**Mean**	**SD**	**Mean**	**SD**	***p*** **-value**	**Mean**	**SD**	**Mean**	**SD**	***p*** **-value**
**Years of working (*****n*** **=** **276)**	9.7	3.6	10.3	4.1	0.521	9.9	3.9	10.1	3.5	0.448	9.9	3.9	10.1	3.3	0.542
**Working hours per day (*****n*** **=** **264)**	8.2	0.6	8.4	1.2	0.074	8.2	0.8	8.4	1.1	0.331	8.2	0.8	8.5	1.1	0.179

Table 3 shows the characteristics of neuropathic pain by using the DN4 questionnaire. The most commonly reported symptom associated with neuropathic pain was limb numbness, followed by sensation of ant crawling, and electric shock. Furthermore, the people who reported spinal pain or osteoarthritis pain, or both types of pain self-reported a higher prevalence of having neuropathic pain signs (based on the DN4 instrument) compared to those who reported no pain. There were statistically significant differences in DN4 scores between people having spinal pain (1.2 ± 1.0) and no spinal pain group (0.2 ± 0.5); people having osteoarthritis pain (1.2 ± 1.0) and no pain group (0.4 ± 0.8) with *p* < 0.05; and people having both spinal pain and osteoarthritis pain (1.5 ± 0.9) and no pain group (0.4 ± 0.8).

**Table 3:** Characteristics of neuropathic pain among participants.

**Characteristics**	**Spinal pain**					**Osteoarthritis pain**					**Spinal pain and osteoarthritis pain**				
	**No**		**Yes**		***p*** **-value**	**No**		**Yes**		***p*** **-value**	**No**		**Yes**		***p*** **-value**
	***n***	**%**	***n***	**%**		***n***	**%**	***n***	**%**		***n***	**%**	***n***	**%**	
Pricking, tingling pins, needles	6	3.8	26	21.8	<0.001	20	9.6	12	17.9	0.858	21	9.1	11	23.9	0.063
The feeling of electric shock in the limbs	2	1.3	13	10.9	<0.001	10	4.8	5	7.5	0.286	11	4.8	4	8.7	0.286
Feeling hot, burning hands and feet	0	0.0	7	5.9	0.002	5	2.4	2	3.0	0.539	5	2.2	2	4.3	0.539
Feeling numbness in the limbs	14	8.9	51	42.9	<0.001	29	13.9	36	53.7	<0.001	36	15.7	29	63.0	<0.001
Itching	1	0.6	7	5.9	0.013	4	1.9	4	6.0	0.101	5	2.2	3	6.5	0.101
Painful cold or freezing pain	2	1.3	9	7.6	0.009	5	2.4	6	9.0	0.027	6	2.6	5	10.9	0.027
Hypoesthesia to touch	0	0.0	7	5.9	0.002	2	1.0	5	7.5	0.010	2	0.9	5	10.9	0.010
Hypoesthesia to prick	1	0.6	9	7.6	0.003	3	1.4	7	10.4	0.002	3	1.3	7	15.2	0.002
Brushing	0	0.0	8	6.7	0.001	5	2.4	3	4.5	0.302	5	2.2	3	6.5	0.302
**Neuropathic pain**	1	0.6	2	1.7	0.397	1	0.5	2	3.0	0.147	2	0.9	1	2.2	0.147
	**Median**	**IQR**	**Median**	**IQR**	***p*** **-value**	**Median**	**IQR**	**Median**	**IQR**	***p*** **-value**	**Median**	**IQR**	**Median**	**IQR**	***p*** **-value**
**DN4 score**	0	0–0	1	0–2	<0.001	0	0–1	1	0–2	<0.001	0	0–1	1	1–2	<0.001

Factors associated with the spinal pain, osteoarthritis pai n, and neuropathic pain of the workers are presented in Table 4. In terms of work conditions, people maintaining constant posture when working from 30 to 60 min (OR = 3.15, 95% CI = 1.07; 9.29), or over 60 min (OR = 2.59; 95% CI = 1.12; 5.98) had a higher odds of suffering from spinal pain. People who worked in a lack of light conditions were also likely to be suffering from osteoarthritis pain with OR = 4.26, 95% CI = 1.02; 17.74. In terms of DN4 score, working conditions including having noise (Coef. = 1.23; 95% CI = 1.06; 1.43), lack of light (Coef. = 1.75; 95% CI = 1.18; 2.61), wet (Coef. = 0.82; 95% CI = 0.77; 0.87), and toxic chemicals (Coef. = 1.93; 95% CI = 1.49; 2.50) had negatively associated with the level of neuropathic pain.

**Table 4:** Associated factors related to the spinal pain, osteoarthritis pain, and neuropathic pain of the workers.

**Characteristics**	**Spinal pain (Yes vs. No)**		**Osteoarthritis pain (Yes vs. No)**		**Spinal pain and osteoarthritis pain (Yes vs. No)**		**DN4 score**	
	**OR**	**95% CI**	**OR**	**95% CI**	**OR**	**95% CI**	**Coef**.	**95% CI**
**Socio-demographic characteristics**								
**Gender (Female vs. Male)**	4.89^*^	0.74; 32.32	0.94	0.16; 5.43	3.29	0.40; 27.31	0.78^*^	0.57; 1.05
**Age (years)**	0.92	0.79; 1.06	1.14	0.95; 1.35	1.26^**^	1.01; 1.58	0.98^*^	0.96; 1.00
**Education (vs. Under high school)**								
High school	2.73	0.56; 13.33	1.50	0.26; 8.74	1.60	0.21; 12.10	1.25^*^	0.97; 1.61
Tertiary or higher	3.99	0.70; 22.67	1.00	0.15; 6.53	1.07	0.12; 9.46	1.04	0.49; 2.19
**Marital status (Living with spouse/partner vs. others)**	0.15	0.01; 1.87	1.43	0.15; 13.62	0.31	0.01; 8.13	0.74	0.48; 1.13
**Number of children (Vs. None)**								
One	1.73	0.20; 15.03	4.88	0.35; 67.93	3.42	0.19; 61.20	1.53	0.63; 3.72
Two	1.35	0.18; 10.16	5.13	0.41; 64.93	4.37	0.26; 73.54	1.49	0.84; 2.62
Three or above	0.78	0.09; 6.99	4.93	0.32; 75.59	3.05	0.14; 66.86	1.63	0.91; 2.92
**Expenditure for health care per year (per USD)**	1.01^***^	1.00; 1.01	1.24	0.81; 1.87	1.42	0.86; 2.37	1.00	1.00; 1.00
**Periodic health examination in company (Yes vs. No)**	1.98	0.40; 9.68	1.62	0.33; 7.81	1.90	0.29; 12.29	1.16	0.90; 1.51
**Health conditions and health behaviors**								
**Having acute symptoms (Yes vs. No)**	1.75	0.82; 3.71	1.87	0.77; 4.54	1.54	0.53; 4.44	0.98	0.68; 1.41
**Having chronic diseases (Yes vs. No)**	1.85	0.87; 3.94	1.33	0.56; 3.14	1.51	0.53; 4.31	1.29	0.87; 1.92
**Having alcohol consumption (Yes vs. No)**	3.65	0.57; 23.53	1.34	0.24; 7.56	3.14	0.39; 25.18	0.89	0.72; 1.10
**Work conditions**								
**Years of experience (per year)**	1.05	0.92; 1.19	0.89	0.74; 1.07	0.75^**^	0.60; 0.95	1.01	0.97; 1.05
**Working hours per day (per hour)**	1.30	0.80; 2.09	0.75	0.41; 1.37	0.78	0.39; 1.59	0.96	0.91; 1.02
**The amount of time maintaining constant posture when working (vs**. <**30 min)**								
30–60 min	3.15^**^	1.07; 9.29	1.37	0.37; 5.07	0.91	0.18; 4.45	0.87	0.70; 1.07
Over 60 min	2.59^**^	1.12; 5.98	2.01	0.77; 5.27	1.51	0.48; 4.74	1.26	0.68; 2.35
**Exposure factors (Yes vs. No)**								
Noise	1.01	0.39; 2.65	1.82	0.53; 6.25	2.56	0.48; 13.78	1.23^***^	1.06; 1.43
Dust	0.74	0.30; 1.84	0.94	0.33; 2.67	0.73	0.22; 2.43	0.99	0.93; 1.06
Hot	0.93	0.35; 2.46	0.35	0.08; 1.52	0.16^**^	0.03; 0.94	0.79	0.48; 1.30
Poisonous gas	0.84	0.33; 2.15	0.45	0.15; 1.33	0.78	0.22; 2.76	0.74	0.44; 1.22
Lack of light	3.80^*^	0.91; 15.82	4.26^**^	1.02; 17.74	3.48	0.71; 17.16	1.75^***^	1.18; 2.61
Wet	0.48	0.12; 1.90	0.60	0.15; 2.35	0.79	0.17; 3.66	0.82^***^	0.77; 0.87
Toxic chemicals	2.30^*^	0.87; 6.06	1.90	0.65; 5.62	1.63	0.49; 5.49	1.93^***^	1.49; 2.50
Working in an accident-prone environment	1.64	0.58; 4.69	1.36	0.46; 3.97	1.22	0.36; 4.14	0.98	0.65; 1.47

^***^p < *0.01*, ^**^p < *0.05*, ^*^p < *0.1*.

## Discussion

Our results highlighted low levels of neuropathic pain but high levels of pain among industrial workers, most notably 43.1% of spinal pain, 24.3% of osteoarthritis pain, and 16.7% of both spinal and osteoarthritis pain. In terms of working conditions, noise, dust and heat are the most common and correlated factors. Maintaining posture for more than 30 min or exposure to above risk factors are associated with higher risks of both spinal pain and osteoarthritis pain.

Within our cohort of Vietnamese factory workers, the prevalence of neuropathic pain was estimated to be 1%, which is low compared to estimates of global prevalence (10). Specifically, some previous studies reported that the prevalence of neuropathic pain in Canada was 17.9% (23), Brazil was 10% (24), French was 6.9% (25), the UK was 8% (26), Australia was 3.3% (11, 23, 27), Brazil (24), was 10%, Thailand was 24.0% (28), and Taiwan was 7.9% (29). This figure may have been underestimated due to various external factors. For instance, despite the fact that our interviews were confidential, workers may have felt that self-reporting of neuropathic pain may result in unwanted attention from managers and uncalled-for changes in working settings or salaries.

Most common symptoms for spinal pain and osteoarthritis pain identified in our study were numbness in the limbs and feeling of ant crawling, itchy, pins and needles in the limbs. Fortunately, both are only first-stage symptoms of these health conditions and thus are subject to potential favorable outcomes. Although hardly eradicated, it has been found that neuropathic pain can be managed if it is within tolerance and will not pose too substantial impacts on patients' quality of life if diagnosed during its early stage (30). Nevertheless, these symptoms can be easily confused with manifestation of other less serious conditions. For instance, numbness in the limbs can also occur as a result of tiredness or overwork, and thus workers may often overlook these symptoms. On the other hand, maintaining constant posture for more than 30 min was also found to be significantly correlated with higher levels of spinal pain. Previous literatures have found out that numbness in limbs may also resulted from constant stiffness and fixed posture (31). Therefore, it is difficult to precisely trace the cause of numbness in limbs as well as feelings of ant crawling and determine whether it is an indicator of neuropathic pain. The confusion between the onset of neuropathic pain and normal tiredness prevents workers from having early diagnosis and ultimately leads to poor treatment outcomes.

Exposure to toxic chemicals and low-light settings were reported as risk factors in workplace settings. The association between toxic chemical exposure and neuropathic pain has been well documented in previous studies, in which potent neurotoxins such as lead, arsenic, and mercury are commonly found in increased quantities within industrial plants, and pose a significant occupational hazard (32–34). Although no specific evidence was available on the correlation between lighting and pain, it might be the fact that low-lit environments prevent workers from identifying other hazards and thus making them more prone to other hazards. In this study, participants who reported working in accident-prone environments had relatively insignificant correlation with neuropathic pain, which may be attributed to increased vigilance due to one's knowledge of these dangers (35). History of acute or chronic medical conditions was also found to be a risk factor, as also indicated in previous studies that neuropathy is frequently secondary to underlying disease processes (e.g., diabetes, multiple sclerosis) (36).

The provision of regular health examinations was associated with a significantly lower rate of osteoarthritis pain as the number of workers with pain was one third of those who were not. Indeed, various studies have underlined the importance of regular checkups in the outcome of neuropathic pain as it is the most effective tool for timely diagnosis of neuropathic pain symptoms. Worldwide, early diagnosis was highlighted as a key measure in neuropathic pain management (37). It was also found that primary prevention, early diagnosis, extensive reduction of risk factors through lifestyle changes and glycaemic control have substantial impact on delaying progression of neuropathic pain. To improve the quality and systemization of neuropathic pain screening during regular health check-ups in Vietnam, a validated scale should be agreed upon. Globally, the Neuropathic Pain Scale and LANSS Pain Scale are among the most common screening tools with high precision (38, 39). Among industrial workers, these scales are highly effective as their conditions are often independent, unlike patients in post-operative and post-trauma settings with a combination of nociceptive and neuropathic pain (40).

Higher annual expenditure for healthcare was associated with lower rates of spinal pain in our sample. Understandably, spinal pain can be substantially alleviated with pharmaceutical treatments as well as prevention measures. It has been found that the majority of spinal pain patients tend to not seek hospital care until the severe stage of the condition (41). While this trend may be attributed to various factors, however, significant proportion of this causes by lacking of budget for healthcare. Patients with more generous financial capacity for healthcare also tend to attend regular health check-ups and thus are more likely to early diagnose and receive timely treatments for this condition, resulting in lower rates of spinal pain.

Several implications can be drawn fr om our study. First, a more logical working scheme should be provided to avoid staying in one posture for more than 30 min. Secondly, more effective occupational health and safety regulation and standards may help to protect workers against workplace exposure to neurotoxins or other occupational safety factors associated with neuropathic development. The adoption of such guidelines has been associated with improvements in health equity and workers' health outcomes in other countries and may serve to improve outcomes among Vietnamese factory workers as well (42). Controlling exposures to hazards in the workplace is vital to protecting workers. Hence, authorities should renovate the lighting system to ensure basic working conditions and prevent further work hazards. While toxic chemicals cannot be completely eliminated due to the nature of industrial work, however, the hierarchy of controls should be applied to reduce and remove hazards (43). Lastly, health examinations should be provided regularly and free-of-charge. In existing workplace health examination programs, a small screening test for spinal and osteoarthritis pain should be included to make early diagnosis of these conditions.

As with any non-randomized observational study, our study contained potential sources of bias, including selection and sampling bias. Firstly, the convenience sample method was applied as well as only workers from three manufacturers were recruited for the study. Hence, our sample could not be representative of the whole Vietnamese industrial worker population. Secondly, only a small cross-section of the workforce was involved, which may impact this study's degree of external validity. Therefore, a cohort study on neuropathic pain among industrial workers in multiple centers could be conducted. Furthermore, occupational exposures including loud noise, dust, hot temperatures, poisonous gas, low-light settings, wet environments, and toxic chemicals were mainly measured by self-reported questionnaires, which might lead to bias and confounders. Lastly, the current study lacked the assessment of several other causes that might lead to neuropathic pain such as diabetes, and central nervous system disorders (Stroke, Parkinson's disease, multiple sclerosis, etc.). Hence, further studies could be conducted to explore the association between these factors and neuropathic pain among the worker population. Notwithstanding these potential shortcomings, however, this study serves as the first assessment of the prevalence as well as associated factors of neuropathic pain in Vietnam and thus provides a valuable addition to the growing field.

## Conclusion

While not showing a high prevalence of neuropathic pain, our study highlighted a high self-reported proportion of industrial workers with spinal and osteoarthritis pain. Notable self-reported factors that were more likely to experience associated with pain were identified, including long time of maintaining one posture, toxic chemicals, and low lighting in working environment. Regular health examination and high budget for healthcare were found be correlated with lower likelihood of neuropathic pain. We proposed several implications, including a new working scheme, improvement of the working environment, provision of regular healthcare services, and adoption of pain screening programs for industrial workers as well as more stringent occupational health and safety standards.

## Data availability statement

The original contributions presented in the study are included in the article/supplementary material, further inquiries can be directed to the corresponding author.

## Ethics statement

The studies involving human participants were reviewed and approved by the Institutional Review Board of Hanoi Medical University (code: 01a-QD/VNCTN). The patients/participants provided their written informed consent to participate in this study.

## Author contributions

Conceptualization: RC, QN, LN, AD, KD, THN, VN, AN, TT, CL, CH, and RH. Data curation: RC, TTN, TTPN, and AD. Formal analysis: QN, LN, KD, THN, VN, TT, CL, and CH. Investigation: LN, TTN, AD, AN, and TT. Methodology: LN and TTN. Project administration: AD, AN, CH, and RH. Resources: TT and CL. Software: TT. Supervision: LN, CL, and RH. Validation: TTN and VN. Writing—original draft: RC, QN, LN, TTN, TTPN, AD, KD, THN, VN, AN, TT, CL, CH, and RH. Writing—review and editing: RC, QN, TTPN, LN, AD, KD, THN, VN, AN, CL, CH, and RH. All authors contributed to the article and approved the submitted version.
